# Intratumoral heterogeneity of programmed cell death ligand-1 expression is common in lung cancer

**DOI:** 10.1371/journal.pone.0186192

**Published:** 2017-10-19

**Authors:** Sayuri Nakamura, Kentaro Hayashi, Yuki Imaoka, Yuka Kitamura, Yuko Akazawa, Kazuhiro Tabata, Ruben Groen, Tomoshi Tsuchiya, Naoya Yamasaki, Takeshi Nagayasu, Junya Fukuoka

**Affiliations:** 1 Department of Pathology, Nagasaki University Graduate School of Biomedical Sciences, Nagasaki, Japan; 2 Department of Surgical Oncology, Nagasaki University Graduate School of Biomedical Sciences, Nagasaki, Japan; Virginia Commonwealth University, UNITED STATES

## Abstract

Programmed cell death ligand-1 (PD-L1) expression may predict the response to both programmed cell death-1 and PD-L1 inhibitors in lung cancer. However, the extent of intratumoral heterogeneity of PD-L1 expression, which may cause false negative results, is largely unexplored. We aimed to assess the intratumoral heterogeneity of PD-L1 expression in surgically resected lung cancer specimens by applying a novel method of tissue microarray, namely Spiral Arrays, which enables us to observe the heterogeneity in spiral-shaped tissue cores. Adenocarcinoma and squamous cell carcinoma specimens were obtained from consecutive patients with lung cancer who had undergone surgical resection at Nagasaki University Hospital (Nagasaki, Japan) since 2009. Small cell lung cancer and large cell carcinoma specimens were selected from patients in the same archive who had undergone resection since 1998. Spiral Arrays were constructed of spiral-shaped cores, prepared from representative blocks of each case, which were subjected to immunohistochemistry using an anti-PD-L1 antibody. Each core was divided into 8 segments and each segment was classified as either PD-L1-positive or PD-L1-negative using thresholds of 1.0%, 5.0%, 10.0%, and 50.0%, respectively. In total, 138 specimens were selected, including 60 adenocarcinomas, 59 squamous cell carcinomas, 12 small cell lung cancers, and 7 large cell carcinomas. The majority of specimens with PD-L1-positive segments exhibited heterogeneous expression (i.e., had a mixture of PD-L1-positive and PD-L1-negative segments within a core) irrespective of the threshold (1.0%, 66.7%; 5.0%, 74.4%; 10.0%, 75.8%; and 50.0%, 85.7%]. Large variations in the ratios of PD-L1-positive segments were observed. At least 50.0% of the segments within a core were negative in no fewer than 50.0% (range, 50.0–76.0%) of cases with heterogeneous PD-L1 expression. In conclusion, intratumoral heterogeneity of PD-L1 expression was frequently observed in cases of lung cancer. Thus, multiple tumor biopsy specimens may be needed to accurately determine the PD-L1 expression status.

## Introduction

Lung cancer is the leading cause of cancer-related mortality. The 5-year relative survival rate is 10.0–15.0% worldwide [1] and is currently 29.7% in Japan [2]. Although, during the last few decades, patients with lung cancer have been treated with a variety of tailored therapeutic strategies (e.g., according to histological type or gene expression profiles) [3, 4], survival still remains poor. Recently, immunotherapy targeting immune checkpoint molecules, especially programmed cell death-1 and programmed cell death ligand-1 (PD-L1), have been approved by the United States Food and Drug Administration for the treatment of patients with advanced non-small cell lung cancer (NSCLC), and are changing the paradigm for therapy in lung cancer [5–7]. At the same time, the assessment of PD-L1 expression using immunohistochemistry (IHC) has become important as a biomarker for predicting response to these therapies [8, 9]. However, previous studies have reported a broad range of PD-L1 expression in NSCLC, ranging from 7.4% to 72.7% of cases [10, 11]. Furthermore, a therapeutic response has been observed not only in patients classified as PD-L1-positive from IHC, but also in some patients classified as PD-L1-negative from IHC, indicating the potential for insufficient sampling to have occurred from the PD-L1-positive region. Some studies have reported the presence of intratumoral heterogeneity of PD-L1 expression in lung cancer. However, the rate and characteristics of the heterogeneity remain largely unexplored [12–15].

In the present study, we aimed to assess the intratumoral heterogeneity of PD-L1 expression in surgically resected lung cancer specimens by employing a unique tissue microarray technique, Spiral Arrays, which enables us to observe the heterogeneity in spiral-shaped tissue cores [16–18].

## Materials and methods

### Ethical statement

The study protocol was approved by the Ethical Review Board Committee (approval number: 16072526) of Nagasaki University (Nagasaki, Japan) on July 26, 2016. Informed consent was obtained from each patient at the time of surgery.

### Tissue specimens

Adenocarcinoma and squamous cell carcinoma specimens were prospectively obtained from consecutive patients with lung cancer who had undergone surgical resection at Nagasaki University Hospital (Nagasaki, Japan) since 2009. Small cell lung cancer and large cell carcinoma specimens were also selected from patients in our institutional archive, but who had undergone surgical resection since 1998, because of the low number of cases due to the infrequency of these histological types. Pathological reports were reviewed and patients with only one of these histological components were included. Hematoxylin and eosin (H&E)-stained slides were also reviewed, and patients with insufficient numbers of malignant cells to construct the Spiral Arrays were excluded. Patients lacking sufficient formalin-fixed, paraffin-embedded tissue were also excluded.

### Spiral Array construction

**Fig 1:**
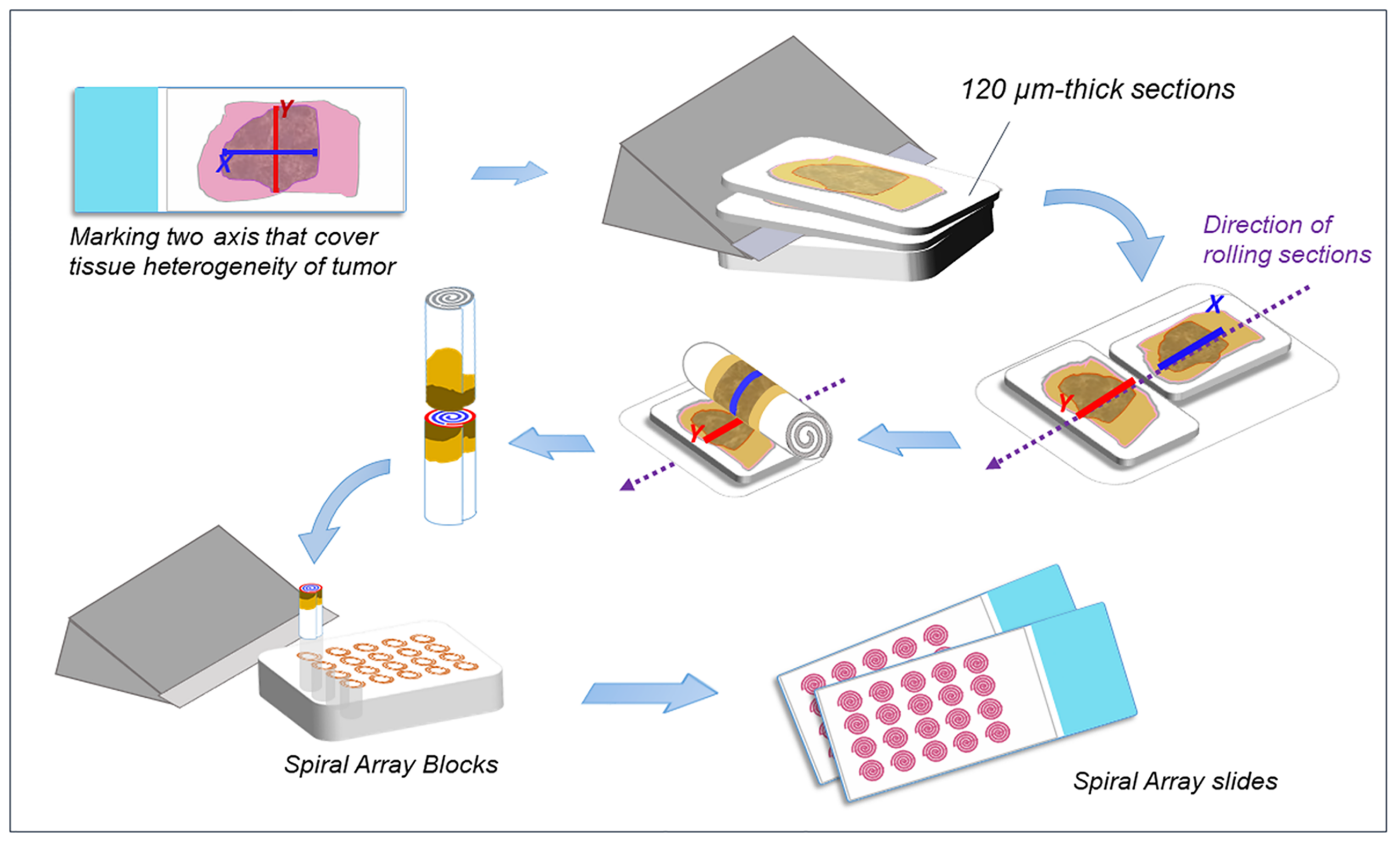
Spiral Array construction. A representative block was selected from each case, and two continuous straight regions of interest (X and Y axes) were selected and marked by reviewing hematoxylin and eosin-stained slides. Two 120.0-μm-thick sections were sliced from each block and aligned with the X and Y axes. The two sections were rolled together into a cylindrical form and cut along the line reflecting the X and Y axes. Spiral Array cores were embedded vertically into a recipient block. Four-μm-thick sections were prepared from the Spiral Array blocks for further histopathological evaluation.

Spiral Arrays were constructed as described previously (Fig 1) [17]. Briefly, single blocks of tissue with the most representative tumor histology and of sufficient quantity was selected from each case. The corresponding H&E-stained slide was digitally scanned using a ScanScope^®^ Aperio CS2 slide scanner (Leica Microsystems, Melbourne, Australia). The scanned whole-slide image of each H&E-stained slide was reviewed to select and mark two continuous straight regions of interest (X and Y axes) prior to constructing the Spiral Arrays. Two 120.0-μm-thick sections were subsequently cut from each block and arranged together on the Spiral Array Constructor (Sakura Finetek Japan Co., Ltd., Tokyo, Japan) as the X or Y axis on each section was aligned in the same direction. The aligned sections were rolled together into a cylindrical form and cut along the line reflecting the X and Y axes. One of the separated reels was embedded vertically into a recipient block to construct the Spiral Arrays. Finally, 4.0-μm-thick sections were prepared from the Spiral Array blocks for further histopathological evaluation using H&E staining and IHC.

### Antibody selection and immunohistochemistry

Well-characterized anti-PD-L1 (clone 28–8; Abcam, Cambridge, MA, USA) and anti-cluster of differentiation 68 (CD68) (clone KP1; DAKO, Glostrup, Denmark) antibodies were selected. IHC was performed using an automated staining system (Leica Bond III; Leica Microsystems). The antibody dilutions were optimized to 1:100 for anti-PD-L1 and 1:400 for anti-CD68. The slides were dewaxed and rehydrated using distilled water, and were subsequently processed for PD-L1 (heat-induced antigen retrieval at pH 9.0) or CD68 (proteolytic treatment). After incubation with the primary antibodies (anti-PD-L1, 30 minutes; anti-CD68, 15 minutes), the tissue sections were rinsed, and the sections for PD-L1 staining were further incubated with EnVision FLEX+ Rabbit LINKER (DAKO) and EnVision+ HRP Labelled Polymer (DAKO). The sections for CD68 staining were incubated with the Bond Polymer Refine Detection Kit (Leica Microsystems). Staining was visualized using diaminobenzidine, and counterstaining was performed using hematoxylin. Formalin-fixed, paraffin-embedded tissue blocks of human placenta and tonsil were prepared as positive controls. The stained slides were scanned as whole-slide images using a ScanScope^®^ Aperio CS2 slide scanner (Leica Microsystems).

### PD-L1 immunohistochemistry scoring on the Spiral Array

PD-L1 expression was only evaluated in tumor cells. We attempted to exclude intratumoral immune cells, such as macrophages and lymphocytes. PD-L1 positivity was defined as any tumor cell that expressed PD-L1 on the cell membrane at any intensity. The tumor cells’ PD-L1 positivity was scored using four different positivity thresholds (≥1.0%, ≥5.0%, ≥10.0%, and ≥50.0%) in any tumor that included a minimum of 100 tumor cells.

**Fig 2:**
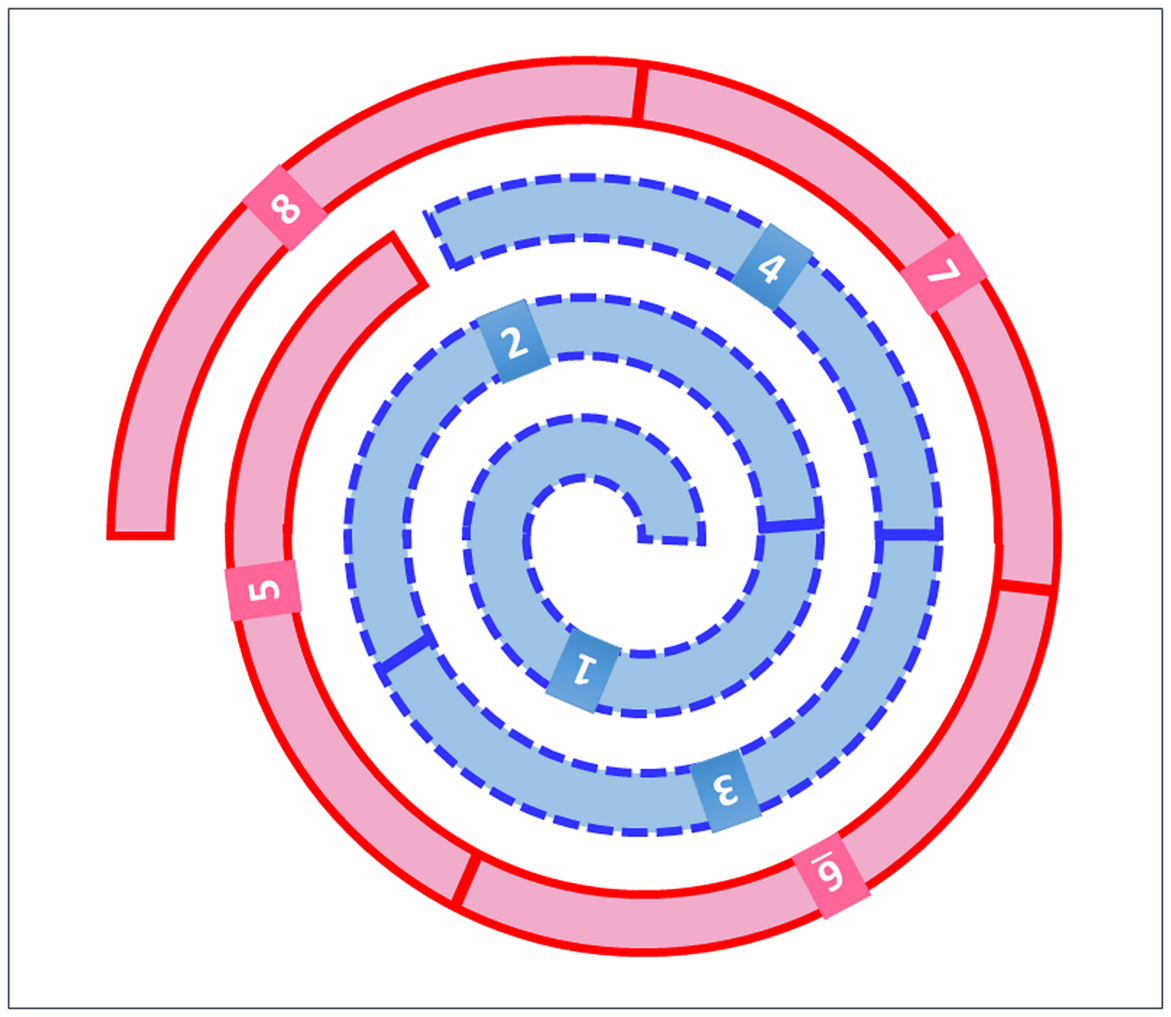
Schematic of the 8 segments of a Spiral Array core. Each core was divided into 8 segments for the scoring of PD-L1 expression.

To evaluate tissue heterogeneity, each Spiral Array core was divided into 8 segments as shown in Fig 2, and positive or negative PD-L1 expression was scored for each segment. Scoring was performed by two or three independent observers. Segments with interobserver disagreement were subsequently evaluated at a meeting between the independent observers and a pulmonary expert, and the final classification was selected after a consensus was reached. Cases with both positive and negative segments within the Spiral Array core were defined as having heterogeneous PD-L1 expression, while cases with identical results for all 8 segments (positive/negative) were defined as having homogeneous PD-L1 expression.

### Comparison between Spiral Arrays and whole tissue sections

Six cases were randomly selected from those with positive PD-L1 staining in the Spiral Array. The staining heterogeneity was compared between the Spiral Array core and the whole tissue section in each case.

### Statistical analyses

The patients’ characteristics at diagnosis were presented as the frequency and percentage for categorical data, and as the median and interquartile range for continuous data. Interobserver agreement for PD-L1 IHC scoring was assessed using the kappa statistic for all possible pairs among the three observers. An average kappa statistic was also calculated for each value [19, 20].

Descriptive and comparative analyses were performed using Microsoft Excel 2010 (Microsoft Corporation, Redmond, WA, USA) and JMP software for the patients’ characteristics (version 11; SAS Institute Inc., Cary, NC, USA). The kappa statistics were calculated using Microsoft Excel 2010 (Microsoft Corporation) and Easy R software (Saitama Medical Center, Jichi Medical University, Saitama, Japan) [21], a graphical user interface for R (The R Foundation for Statistical Computing, Vienna, Austria).

## Results

### Patient characteristics

The patients’ clinicopathological characteristics are shown in Table 1. Among the 138 cases of lung cancer, we identified 60 cases of adenocarcinoma, 59 cases of squamous cell carcinoma, 12 cases of small cell lung cancer, and 7 cases of large cell carcinoma. The majority of patients were male (73.9% *vs*. 26.1%), and the median age was 70 (range, 41–90) years. The majority of patients were current or former smokers (18.1% and 57.3%, respectively). The median smoking index was 900 (range, 0–3,220). Almost all of the patients had Stage I, II, or IIIA disease (59.4%, 22.5%, and 13.8%, respectively).

**Table 1:** Patient characteristics.

Characteristics	Patients (n = 138)
Sex, n (%)	
Male	102 (73.9)
Female	36 (26.1)
Age (years), median (range)	70 (41–90)
Smoking status, n (%)	
Non-smoker	33 (23.9)
Ex-smoker	79 (57.3)
Smoker	25 (18.1)
Unknown	1 (0.7)
Smoking index, median (range)	900 (0–3,220)
Histology, n (%)	
Adenocarcinoma	60 (43.5)
Squamous cell carcinoma	59 (42.7)
Small cell lung cancer	12 (8.7)
Large cell carcinoma	7 (5.1)
Stage, n (%)	
I	82 (59.4)
II	31 (22.5)
IIIA	19 (13.8)
IIIB	2 (1.4)
IV	4 (2.9)
Tumor size (mm), median (range)	25.0 (9.0–100.0)
Tumor status, n (%)	
T1	61 (44.2)
T2	60 (43.5)
T3	13 (9.4)
T4	4 (2.9)
Node status, n (%)	
N0	101 (73.2)
N1	19 (13.8)
N2	16 (11.6)
N3	2 (1.4)

### PD-L1 expression and heterogeneity

**Fig 3:**
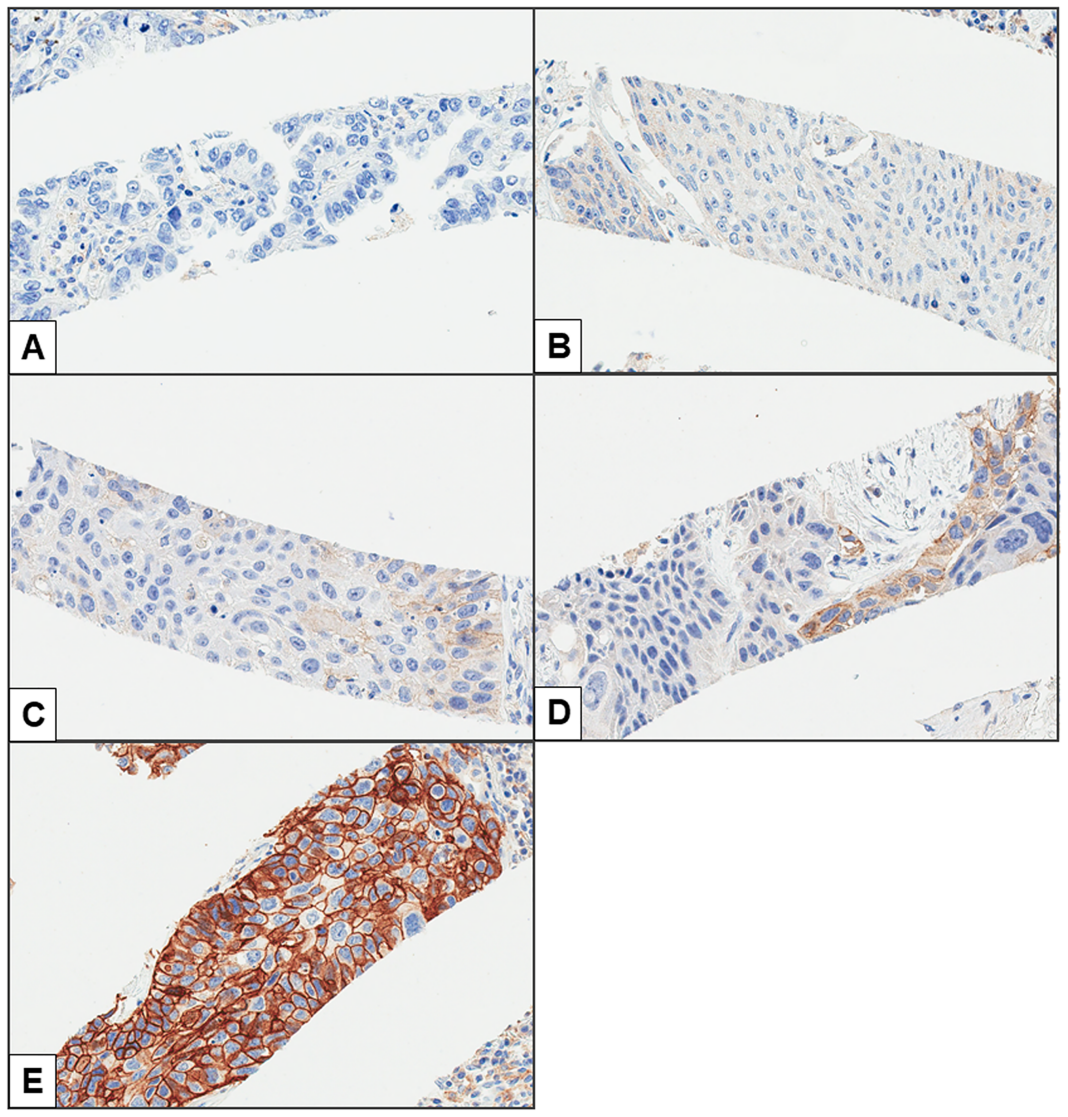
Representative images of PD-L1 expression. (**A**) <1.0%, (**B**) 1.0–4.9%, (**C**) 5.0–9.9%, (**D**) 10.0–49.9%, and (**E**) ≥50.0% PD-L1-positive cells (magnification, 200×).

**Fig 4:**
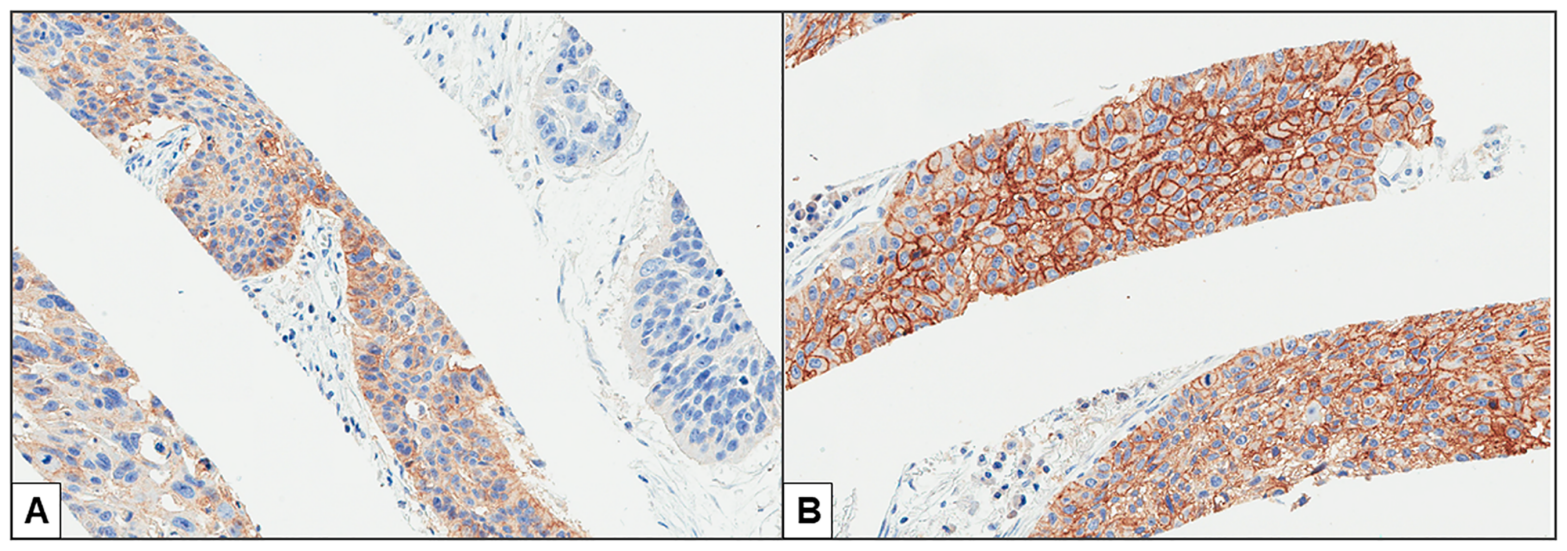
Representative images of the heterogeneous and homogeneous expression of PD-L1. (**A**) Heterogeneous and (**B**) homogeneous expression of PD-L1 (magnification, 150×).

**Fig 5:**
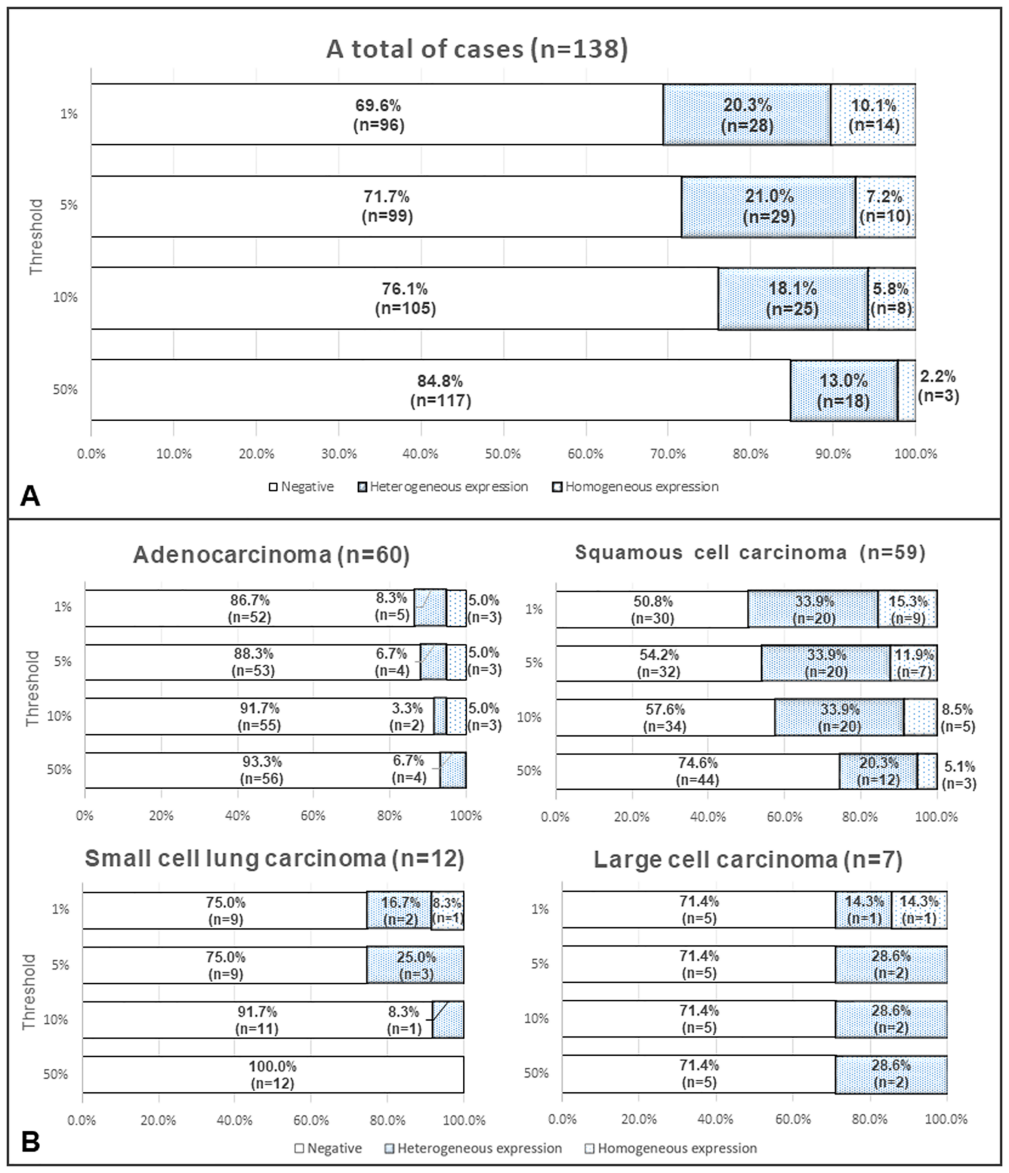
Distributions of cases with negative, heterogeneous, and homogeneous PD-L1 expression. (**A**) Distributions of all 138 cases and (**B**) distributions according to histological type.

**Fig 6:**
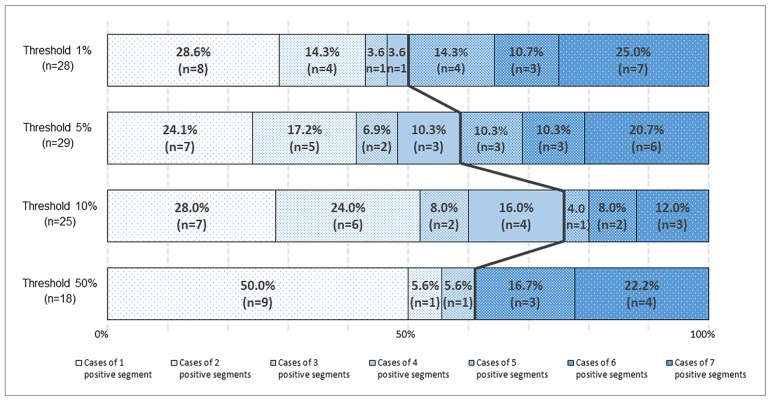
Distributions of cases with heterogeneous PD-L1 expression and varying numbers of PD-L1-positive segments. The left side of the black line indicates the proportions of cases where negative segments account for ≥50.0% of the total number of segments within the cores and the right side of the black line indicates the proportions of cases where positive segments account for >50.0% of the total number of segments within the cores. At any of the thresholds, ≥50.0% of the total number of segments within the cores were negative in no fewer than 50.0% (range, 50.0–76.0%) of cases with heterogeneous PD-L1 expression.

**Fig 7:**
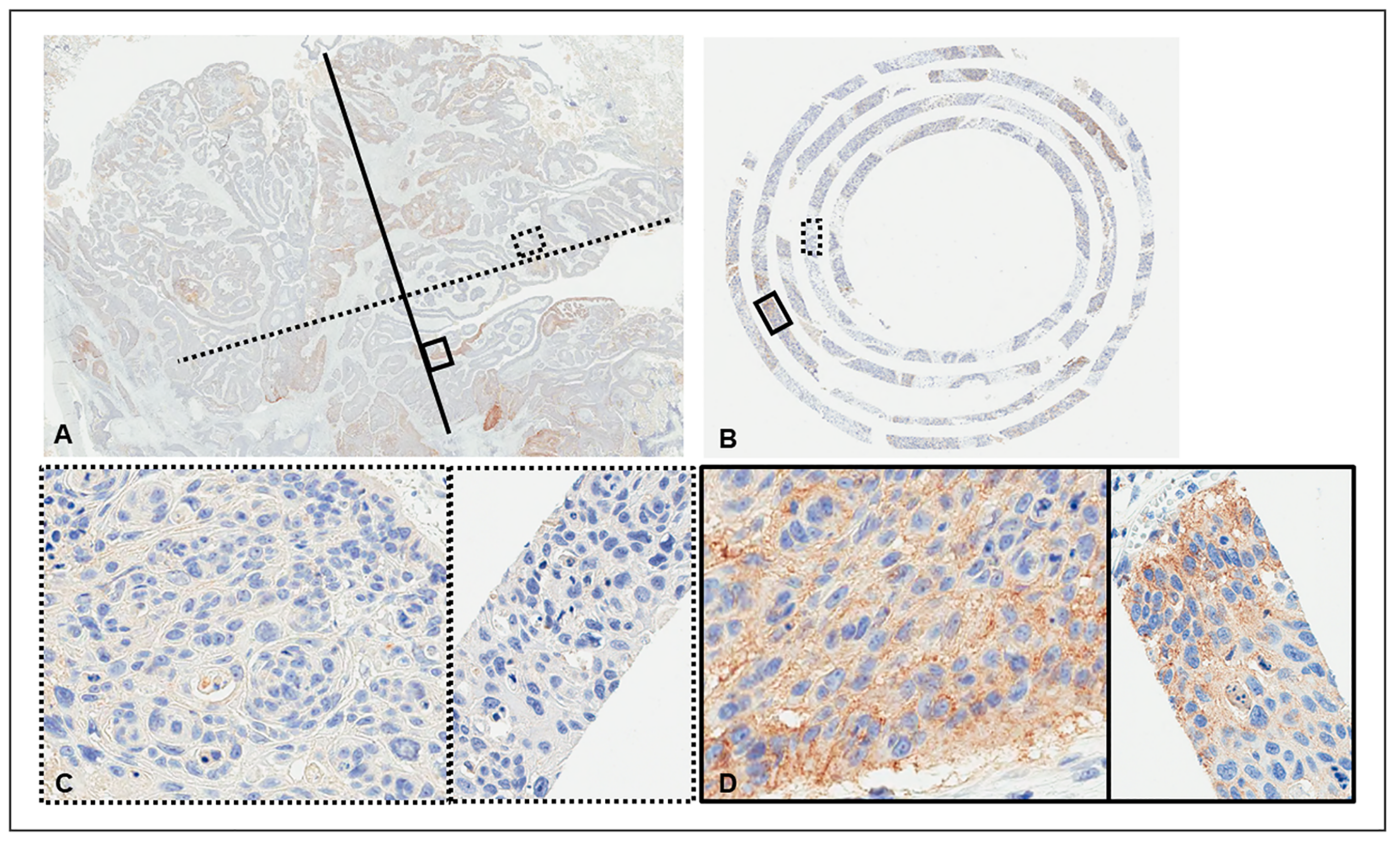
Representative images of PD-L1 expression in a case with heterogeneous PD-L1 expression. (**A**) The whole section (magnification, 3×), (**B**) the corresponding Spiral Array core (magnification, 15×), (**C**) magnified images from the regions delineated by the dotted lines in **A** (left) and **B** (right) (magnification, 200×), and (**D**) magnified images from the regions delineated by the solid lines in **A** (left) and **B** (right) (magnification, 200×). Images of the other 5 cases are shown in S1 Fig.

Representative images of PD-L1 expression for each criterion are shown in Fig 3A–3E. Representative cores with and without heterogeneous PD-L1 expression are shown in Fig 4A and 4B. The distributions of cases with negative, heterogeneous, and homogeneous PD-L1 expression are shown in Fig 5A and 5B. The threshold-specific number of cases with ≥1 PD-L1-positive segment was 42 (30.4%), 39 (28.3%), 33 (23.9%), and 21 (15.2%) for the 1.0%, 5.0%, 10.0%, and 50.0% thresholds, respectively. The majority of cases had heterogeneous PD-L1 expression irrespective of the threshold (1.0%: 66.7% [n = 28], 5.0%: 74.4% [n = 29], 10.0%: 75.8% [n = 25], and 50.0%: 85.7% [n = 18]) (Fig 5A). When cases were categorized according to their histological type, PD-L1-positive segments were observed in 6.7–13.3% of adenocarcinomas, 25.4–49.2% of squamous cell carcinomas, ≤25.0% of small cell lung cancers, and 28.6% of large cell carcinomas, depending on the threshold that was used (Fig 5B). At least 50.0% of the 8 segments within the cores were negative for PD-L1 expression in the majority of cases with heterogeneous PD-L1 expression irrespective of the threshold (range, 50.0–76.0%), although the ratio of PD-L1-positive segments varied considerably among the cases (Fig 6). The heterogeneous and homogenous staining patterns of PD-L1 expression were identical between the Spiral Arrays and whole tissue sections in all 6 randomly selected cases (Fig 7A–7D and S1 Fig). The heterogeneous expression pattern of PD-L1 on the Spiral Arrays was comparable to that of the original whole tissue sections (Fig 7A–7D).

### Interobserver agreement of PD-L1 expression scores

The interobserver agreement for PD-L1 IHC scoring among the three observers is shown in Table 2. In total, 917 segments were scored by the three observers. The number of segments in agreement between the three observers (i.e., positive *vs*. negative) for each threshold (1.0%, 5.0%, 10.0%, and 50.0%) were 814, 824, 850, and 869, respectively. The average kappa statistics were 0.76, 0.75, 0.78, and 0.79 for the 1.0%, 5.0%, 10.0%, and 50.0% thresholds, respectively.

**Table 2:** The kappa statistics for the PD-L1 immunohistochemistry scores for segments (n = 917).

		Threshold			
		1%	5%	10%	50%
Observer1	Observer2	0.72	0.70	0.76	0.80
Observer2	Observer3	0.77	0.72	0.75	0.73
Observer1	Observer3	0.79	0.82	0.83	0.84
Average		0.76	0.75	0.78	0.79

## Discussion

In the present study, using a newly developed tissue microarray method, namely Spiral Arrays, we revealed that intratumoral heterogeneity of PD-L1 expression was common in lung cancer. In past studies, the intratumoral heterogeneity of PD-L1 expression has been reported in solid tumors, including lung cancer, breast cancer, melanoma, and renal cell carcinoma [10, 12–14, 22–29]. However, the majority of studies have evaluated the intratumoral heterogeneity between primary and metastatic sites [14, 26], and only a few studies have evaluated the intratumoral heterogeneity within tumors. McLaughlin *et al*. [12], for instance, have recently evaluated the intratumoral heterogeneity within surgically resected NSCLC tumor specimens using IHC and quantitative fluorescence, and have reported the presence of intratumoral heterogeneity of PD-L1 expression. Furthermore, an autopsy case report revealed that PD-L1 and associated immunogenetic profiles differed according to metastatic site in the same patient [15].

To our knowledge, this is the first study to evaluate the intratumoral heterogeneity of lung cancer using a segmental approach with several thresholds. We used a Spiral Array technique with 8 segmented cores to evaluate intratumoral heterogeneity. Specimens with both PD-L1-positive and PD-L1-negative segments within the cores were defined as having heterogeneous PD-L1 expression. Our findings suggest that PD-L1 frequently exhibits heterogeneity in its expression (Fig 5A). Interestingly, ≥50.0% of the 8 segments within the cores were negative for PD-L1 expression in no fewer than 50.0% of the cases with heterogeneous PD-L1 expression (range, 50.0–76.0%). Thus, cases with heterogeneous PD-L1 expression are more likely to return a false negative result during an assessment that uses small biopsy specimens. Recently, Ilie *et al*. [14] and Kitazono *et al*. [26] compared PD-L1 expression in surgically resected specimens and matched small biopsies from patients with NSCLC. A large difference in the discordant rate (48.0% *vs*. 7.6%) was observed between the two reports. Our findings were comparable to those of Ilie *et al*. [14], suggesting that the apparent PD-L1 status in diagnostic biopsies may not reflect that in resected specimens in many cases. Lung cancer is often only clinically detected in its advanced stages, and a small biopsy is a common primary method for diagnosis and molecular testing. PD-L1 expression is also usually assessed in the clinic using small biopsy specimens [30, 31]. Thus, a higher number of biopsy specimens should be obtained from a single site. However, few studies have evaluated the association between the number of biopsy specimens and biomarker sensitivity.

**Fig 8:**
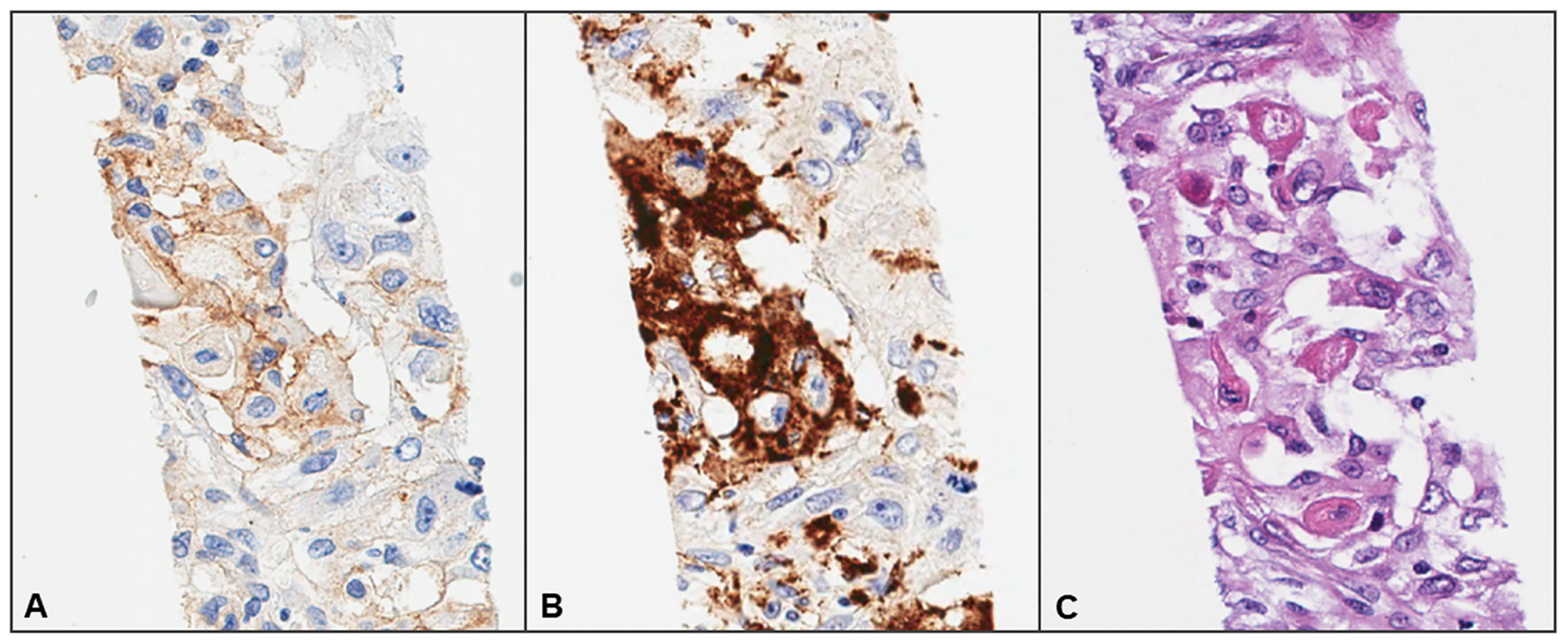
Representative images of a region containing macrophages that resemble tumor cells. Immunohistochemical analysis of (**A**) PD-L1 and (**B**) CD68 expression, and (**C**) hematoxylin and eosin staining (magnification, 200×).

Interobserver agreement regarding PD-L1 expression scores slightly varies according to the thresholds. However, all the thresholds produced good [32]. Disagreement among the three observers may be related to the inadvertent inclusion of immune cells that stain positive for PD-L1 (e.g., dendritic cells or macrophages that infiltrate the tumor nests). Although CD68 IHC was performed to eliminate macrophages, in some instances, it was difficult to differentiate between tumor cells and macrophages (Fig 8A–8C). Thus, we propose that a minimum of two pathologists perform the PD-L1 scoring for each case, and also that CD68 IHC should be used for the assessment of PD-L1 expression in the clinic.

This study has several limitations. First, we retrospectively evaluated surgically resected specimens, and PD-L1 expression is usually assessed in the clinic using small biopsy specimens. Second, we used only one antibody (clone 28–8) against PD-L1 to limit the confusion caused by differences in antigen recognition between different clones. Clone 28–8 has been characterized by several investigators and is used in clinical trials of lung cancer [28, 33–37]. Finally, data were obtained using only the Spiral Array technique, since concordance between the findings from Spiral Arrays and whole tissue sections has been established in a previous study [17].

## Conclusions

Intratumoral heterogeneity of PD-L1 expression was frequently observed in cases of lung cancer. Thus, our findings suggest that a relatively large number of tumor biopsy specimens may be needed to accurately determine PD-L1 expression status.

## Supporting information

S1 Fig Six randomly selected cases with heterogeneous staining between Spiral Arrays and whole tissue sections. Identical staining patterns were observed in all 6 cases.
(PDF)
