# Supplementary material for: Intratumoral heterogeneity of programmed cell death ligand-1 expression is common in lung cancer
Source: PLoS One. 2017 Oct 19;12(10):e0186192. doi: 10.1371/journal.pone.0186192 (PMC5648155; doi:10.1371/journal.pone.0186192)

Adenocarcinoma, case 31

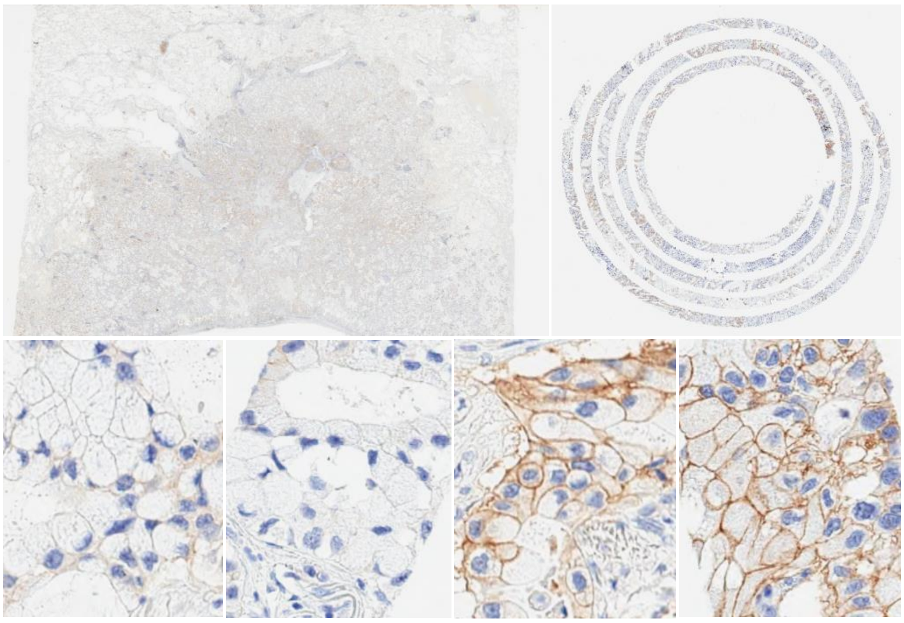

Adenocarcinoma, case 46

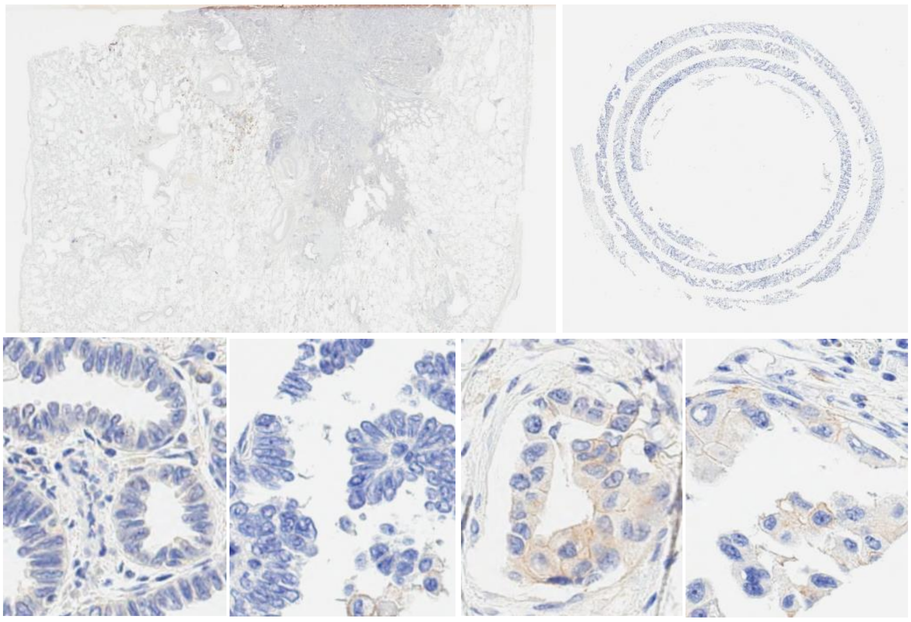

Squamous cell carcinoma, case 5

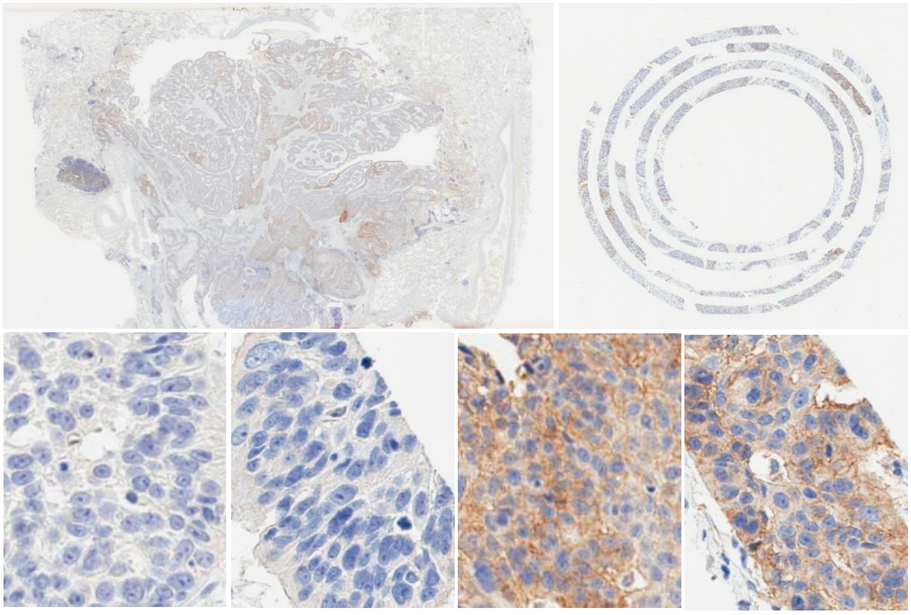

Squamous cell carcinoma, case 21

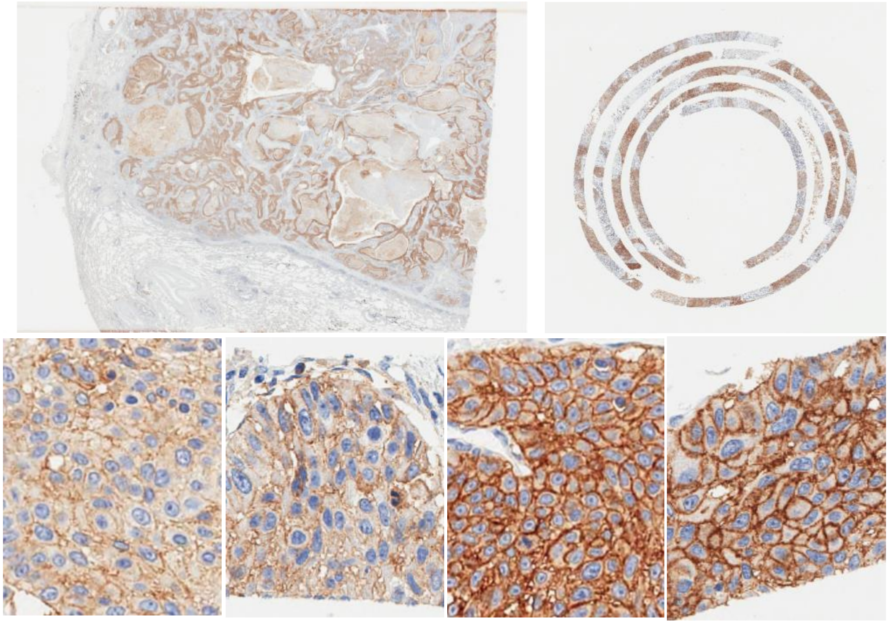

Squamous cell carcinoma, case 31

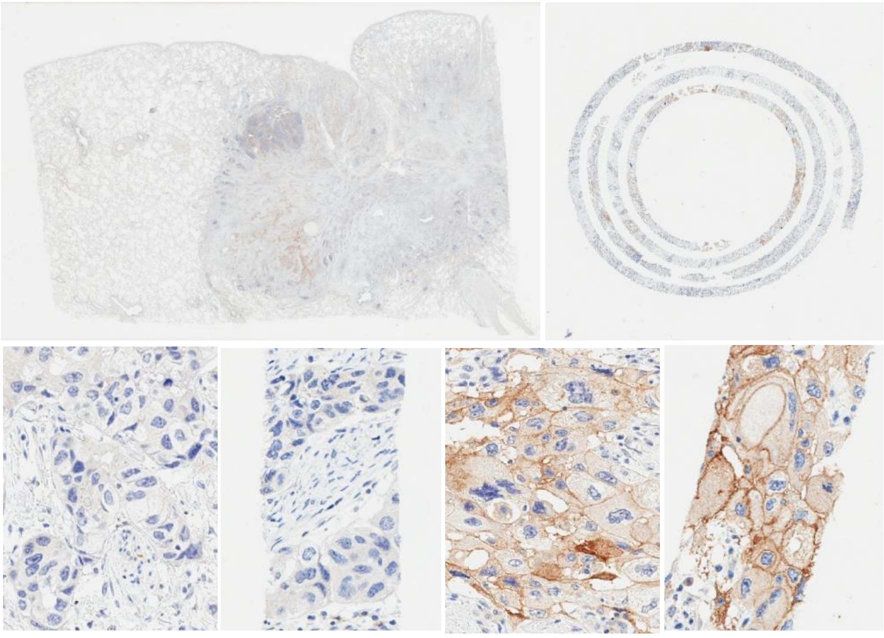

Squamous cell carcinoma, case 53

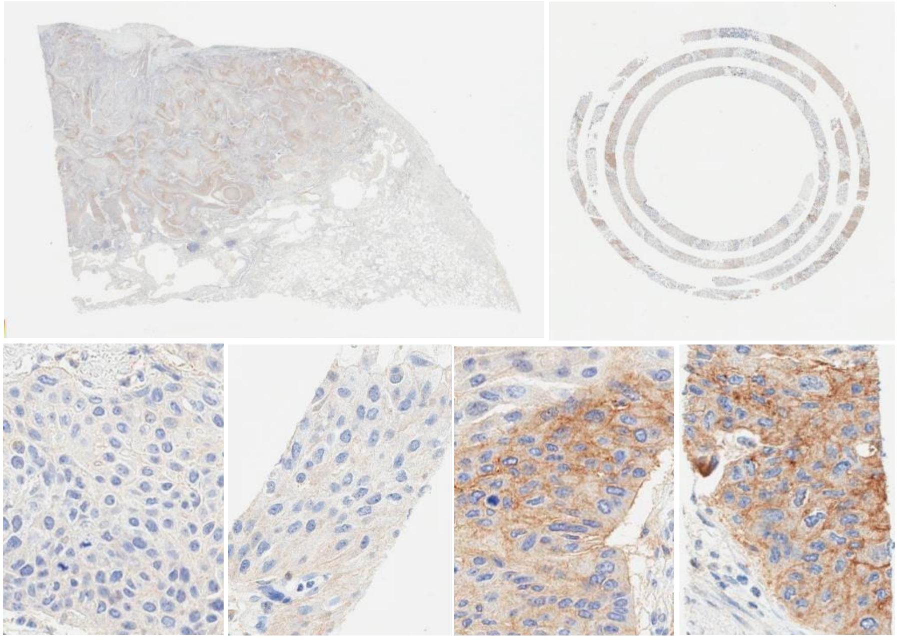

Supplement: S1 Fig — Identical staining patterns were observed in all 6 cases. (PDF) [file pone.0186192.s001.pdf]
